# HPLC Method for Estimation of Metformin Hydrochloride in Formulated Microspheres and Tablet Dosage Form

**DOI:** 10.4103/0250-474X.56031

**Published:** 2009

**Authors:** Mousumi Kar, P. K. Choudhury

**Affiliations:** Department of Pharmaceutical Sciences, Mohan Lal Sukhadia University, Udaipur-313 001, India

**Keywords:** HPLC, metformin hydrochloride, microspheres, recovery studies, tablet

## Abstract

A simple, accurate, economical and reproducible HPLC method has been developed for quantitative estimation of metformin hydrochloride from tablet dosage form and formulated microspheres. The developed HPLC method is a reverse phase chromatographic method using phenomenex C_18_ column and acetonitrile:phosphate buffer (65:35) pH adjusted to 5.75 with o-phosphoric acid as mobile phase and glipizide as internal standard. The linearity was observed in concentration range of 0-25 μg/ml for metformin hydrochloride. Results of analysis were validated statistically and by recovery studies.

Metformin hydrochloride chemically, N,N-dimethylimidodicarbonimidic diamide hydrochloride[1] is an antidiabetic agent[2]. In literature cited for analysis of metformin hydrochloride, few HPLC[3–6] methods have been reported but the retention time has been found to be very high and none have been reported from the microspheres.

A Shimadzu LC-10AT with SPD-10A detector was used for HPLC analysis. The reagents used for preparation of mobile phase were of HPLC grade. High performance liquid chromatographic method was developed using phenomenex C_18_ ODS (5 μ) 250 × 4.60 mm column, mobile phase selected for this method contains acetonitrile:phosphate buffer (65:35) pH adjusted to 5.75 with o-phosphoric acid which was filtered through 0.2 μ membrane filter. Flow rate employed was 1.0 ml/ min. Detection of eluent was carried out at 233.0 nm. Glipizide was used as the internal standard. Column was saturated with mobile phase for about an hour at above specified conditions. After the chromatographic conditions were set and the instrument was stabilized to obtain a steady base line a mixed standard dilution of pure drug containing 10 μg/ml of metformin hydrochloride and 5 μg/ml of glipizide (internal standard) were prepared in mobile phase, filtered through 0.2 μ membrane filter and loaded in the injector of instrument fitted with 20 μl fixed volume loop. The solution was injected three times and chromatogram recorded. The mean retention time for metformin hydrochloride and glipizide were found to be 2.30 and 3.95 min, respectively. The representative chromatogram of metformin hydrochloride and internal standard glipizide is reported in fig. 1.

**Fig. 1:**
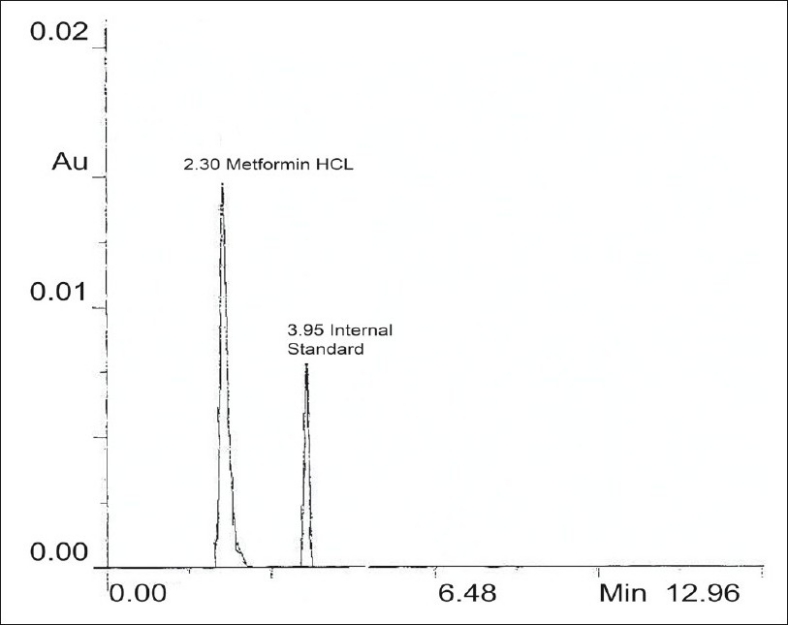
Chromatogram of metformin hydrochloride and the internal standard glipizide

Standard stock solution of metformin hydrochloride and glipizide with concentration of 100 μg/ml was prepared separately in the mobile phase. For preparation of drug solutions for the calibration curve, in a series of 10 ml volumetric flask aliquots of standard stock solution of metformin hydrochloride 0.25, 0.5, 1.0, 1.5, 2.0 and 2.5 ml were transferred and to each flask 0.5 ml of glipizide standard stock solution was added and volume made up to the mark with mobile phase. Each solution was injected after filtration through 0.2 μ membrane filter and a chromatogram was recorded. The calibration curve was plotted between concentration of drug and ratio of peak area of metformin hydrochloride and glipizide (internal standard). Linearity was found to be in concentration range of 0 to 25 μg/ml of metformin hydrochloride with linear regression equation as y= 0.0204x+0.0012 and the correlation coefficient value of 0.9990.

For analysis of formulation, formulated microspheres were accurately weighed and grounded to fine powder. Powder equivalent to 10 mg of metformin hydrochloride was accurately weighed and transferred to a 100 ml volumetric flask containing about 75 ml of mobile phase. The powder mixture was dissolved in the mobile phase with the aid of ultrasonication. The solution was filtered through Whatman filter paper No. 41 into another 100 ml volumetric flask washed the filter paper with mobile phase and added washings to the filtrate. Volume of the filtrate was made up to the mark with the mobile phase. From the above filtrate, 1 ml was taken in a 10 ml volumetric flask to which 0.5 ml of glipizide standard solution (100 μg/ml) was added and volume was made up to the mark with mobile phase, the solution was then filtered through 0.2 μ membrane filter.

After setting the chromatographic conditions and stabilizing the instrument, the formulated microsphere sample solution was injected and a chromatogram was recorded. The injection was repeated three times and the peak area of metformin hydrochloride and glipizide were recorded. The peak area ratio of drug to internal standard was calculated and the amount of drug present was estimated from the respective calibration curve. The analysis of formulated microspheres was compared by repeating the procedure on commercially available tablet formulation of metformin hydrochloride, the result of analysis is reported in Table 1.

**TABLE 1:** RESULTS OF ANALYSIS OF FORMULATED AND COMMERCIAL METFORMIN HYDROCHLORIDE FORMULATION

Batch	Concentration of drug present		% estimated*		Standard Deviation	% Recovery**
				
	Microspheres (mg)	Marketed tablet formulation (mg)	Formulated Microspheres	Marketed tablet formulation				
A	500	500	99.92	99.56	0.124	99.42
B	500	500	99.23	99.78	0.287	100.31
C	500	500	100.28	99.94	0.578	100.11

* Average of five determinations

** Average of determination at three different concentration levels

To study the accuracy, reproducibility and precision of the above methods recovery studies were carried out by addition of standard drug solution to pre-analyzed sample of formulated microspheres at three different concentration levels (5, 10 and 15 μg). Results of recovery studies were found to be satisfactory and are reported in Table 1.

A HPLC method has been developed for estimation of metformin hydrochloride from pharmaceutical dosage form. The developed method for quantitative estimation of metformin hydrochloride from dosage form is reverse phase chromatographic method utilizing C_18_ column and acetonitrile: phosphate buffer as mobile phase. Detection of the eluent was carried out using UV detector. The run time per sample is just 6 min.

The result of analysis of formulated microspheres using this developed method was found close to 100% for metformin hydrochloride, values of standard deviation was satisfactorily low indicating accuracy and reproducibility of the methods. Recovery studies were satisfactory and show that there is no interference of excipients. The developed methods were found to be simple, rapid, and accurate and can be used for routine analysis of metformin hydrochloride from tablet dosage form.
